# Validation of the Japanese version of boredom proneness scale and derivation of its short version among older adults: a cross-sectional study

**DOI:** 10.1186/s12955-026-02490-3

**Published:** 2026-02-04

**Authors:** Atsushi Takayama, Izumi Uehara, Takashi Yoshioka, Taro Takeshima, Kenji Omae, Sayaka Shimizu, Hiroaki Nakagawa, Akihiro Ozaka, Sugihiro Hamaguchi, Shunichi Fukuhara

**Affiliations:** 1 Department of Pharmacoepidemiology, Graduate School of Medicine and Public Health, Kyoto University, Yoshidakonoe-cho, Sakyo-ku, Kyoto, 606-8501 Japan; 2 Institute for Education and Human Development, Ochanomizu University, Tokyo, Japan; 3 Department of Preventive Medicine and Public Health, School of Medicine, Keio University, Tokyo, Japan; 4 Institute of Clinical Epidemiology (iCE), Showa University, Tokyo, Japan; 5 Center for University-Wide Education, School of Health and Social Services, Saitama Prefectural University, Saitama, Japan; 6 Center for Innovative Research for Communities and Clinical Excellence (CiRC2LE), Fukushima Medical University, Fukushima City, Fukushima Japan; 7 Department of Innovative Research and Education for Clinicians and Trainees (DiRECT), Fukushima Medical University Hospital, Fukushima City, Fukushima Japan; 8 Section of Clinical Epidemiology, Department of Community Medicine, Kyoto University, Kyoto, Japan; 9 Patient Driven Academic League (PeDAL), Tokyo, Japan; 10 Department of General Internal Medicine, Fukushima Medical University, Fukushima, Japan; 11 Department of General Medicine, Shirakawa Satellite for Teaching and Research (STAR), Fukushima Medical University, Fukushima, Japan; 12 Department of Health Policy and Management, Johns Hopkins Bloomberg School of Public Health (JHSPH), Baltimore, MD USA

**Keywords:** Aged, Boredom, Health-related quality of life, Socioeconomic factors, Validation study

## Abstract

**Background:**

Boredom proneness is a common yet underexplored psychological trait among older adults, linked to depression, loneliness, and reduced quality of life. Despite its clinical relevance, validated instruments for assessing boredom in this population are scarce. This study aimed to validate the Japanese version of the Boredom Proneness Scale (JBPS) and to develop a short form specifically designed for older adults (s-BPSO).

**Methods:**

We conducted two cross-sectional surveys in Japan. The first, a paper-based survey (*n* = 5,382; mean age = 82), targeted community-dwelling older adults aged ≥ 75 years in Sukagawa City. The second, an internet-based national survey (*n* = 4,124; mean age = 66), recruited participants aged ≥ 50 years. Structural validity of the JBPS was assessed via exploratory and confirmatory factor analysis. Reliability was evaluated using internal consistency and test–retest analysis. The s-BPSO was derived using item response theory and validated independently using data from the second survey. Convergent validity was examined through correlations with validated measures of mental and physical health.

**Results:**

The JBPS demonstrated good internal consistency (Cronbach’s α = 0.87) and test–retest reliability (ICC = 0.84). Exploratory factor analysis supported a three-factor structure, though model fit in confirmatory analysis was modest. The JBPS showed strong correlations with depressive symptoms, loneliness, and HRQOL measures. Existing short versions of the BPS performed poorly in this older sample. A newly developed 6-item short version (s-BPSO) based on a two-factor structure exhibited acceptable model fit (CFI = 0.987, RMSEA = 0.056), high internal consistency (α = 0.80), and strong correlation with the full JBPS (*r* = 0.86). Higher boredom scores were associated with older age, lower income and education, reduced IADL, and lower HRQOL.

**Conclusions:**

The JBPS and s-BPSO are valid and reliable instruments for assessing boredom proneness among older Japanese adults. The s-BPSO provides a concise alternative for use in epidemiological surveys and clinical practice. These tools may inform interventions aimed at promoting active and mentally healthy aging, and support future cross-cultural research on aging-related psychological traits.

**Supplementary Information:**

The online version contains supplementary material available at 10.1186/s12955-026-02490-3.

## Introduction

Boredom, often viewed as a trivial yet pervasive mental state, may signal a loss of an individual’s sense of life’s meaning [1]. The boredom here refers to a chronic boredom tendency, i.e. boredom trait, not the boredom state one feels when doing simple tasks. Since this is a critical component of well-being [2], discussing boredom is essential for successful aging [3, 4]. Indeed, boredom proneness is a recognized predictor of depression [5–8], anxiety [7, 9], loneliness [8, 10, 11], and social isolation [12], —issues that are particularly pertinent to older adults. In the later stage of life, older adults often face challenges such as physical decline [13, 14], loss of independence [15], and the passing of friends or loved ones [16, 17], which may increase their susceptibility to chronic boredom.

Boredom typically arises from a lack of life’s meaning, interest, and challenge and is believed to motivate changes in behavior to address these deficiencies. The Boredom Proneness Scale (BPS) [18] is a self-reported questionnaire, originally developed in English and widely used globally [19–22]. The BPS evaluates individuals’ connection to their environments and their ability to access resources necessary for developing a sense of mastery [18, 22]. Although boredom proneness has been initially recognized as relevant to older adults, research discussions have predominantly focused on younger populations [23, 24]. However, recent studies have highlighted the need for a more in-depth investigation into boredom proneness among older adults [25].

Despite its importance, evidence regarding boredom proneness among older adults remains scarce. Understanding the impact of boredom on this population requires reliable and practical measurement tools. The BPS has recently been translated into Japanese by Uehara, one of the authors of this paper, and is currently being assessed for reliability and validity in adults aged 30–50, independent of this study. However, it has not yet been published, and its reliability and validity among older adults remain unexamined.

For further understanding of successful aging, assessing boredom proneness is particularly significant in Japan, a country with an advanced aging population [26, 27]. Older adults may exhibit unique characteristics distinct from the general adult population, necessitating a separate examination of boredom proneness in this demographic. Additionally, the original BPS consists of 28 items, and several shortened versions have been developed for younger populations. However, no validated short version has been specifically designed for older adults. Therefore, the three objectives of our study were (1) to develop and validate the JBPS, (2) to create and validate the s-BPSO, and (3) to describe how boredom proneness is distributed among older adults and how it relates to key demographic and health-related variables such as sex, age, educational status, annual household income, number of household members, Instrumental Activities of Daily Living (IADL), and Health-Related Quality of Life (HRQOL). The third objective was exploratory and descriptive in nature, aiming to provide foundational information for future research and to contextualize boredom proneness as an emerging construct relevant to healthy and active aging.

## Methods

We followed the COSMIN guidelines for reporting measurement properties [28]. The process of Japanese translation and cultural adaptation of the BPS is provided in Supplementary Methods.

### Japanese translation and cultural adaptation

IU (a psychologist) and her team translated the English version of the BPS into Japanese. This version consisted of the original 28 items (#1 to #28), arranged in the same order, with responses measured on a 7-point Likert scale ranging from a minimum of 7 to a maximum of 196. An expert panel consisting of physicians, a psychiatrist, an epidemiologist, and laypeople reviewed the preliminary Japanese version to confirm its content validity, including the naturalness of the language and overall readability. A psychology specialist, who is a fluent English speaker, then back-translated the Japanese version into English. This back-translation was revised repeatedly until it was deemed equivalent to the original version between IU and the psychology specialist.

### Study design and setting

This cross-sectional study utilized data from two different surveys:

[First survey] For the validation of the JBPS and the derivation of s-BPSO, we utilized data from the Sukagawa Study. This prospective cohort study, established in 2017, targeted community-dwelling individuals aged 75 or older in Sukagawa City. The study was a collaboration with Sukagawa City, Iwase General Hospital, and Fukushima Medical University, aiming to promote healthy aging among community-dwelling older adults [29]. Given that the city’s age distribution mirrors that of Japan as a whole, Sukagawa serves as a typical example of a provincial city in Japan [29]. We distributed paper-based questionnaires to all eligible citizens from March 3 to March 31, 2022, following up with reminder letters to non-respondents. Staff from the municipal department assisted participants in filling out the questionnaire, either directly or via telephone for those who encountered difficulties.

[Second survey] For the validation of the s-BPSO, we conducted a nationwide internet survey from January 25 to January 27, 2023, through the Macromill Inc. (Tokyo, Japan) platform. This survey targeted the general population aged 50 years or older, reflecting the latest national population distribution in Japan [30]. This age threshold was chosen to ensure adequate sample size and diversity of age distribution in an online panel, as the feasibility of obtaining a sufficient number of respondents aged 75 years or older alone was uncertain. Subsequent analyses focusing on those aged 75 years and older were additionally conducted to assess the generalizability of findings to the older-old population. Macromill’s online research panel comprises approximately 10 million registered individuals with detailed demographic and socioeconomic information. Recruitment emails were sent to randomly selected eligible panelists. The number of participants for each age (10-year strata) and sex (men and women) was controlled based on the proportion of the latest official population statistics in Japan. The survey automatically restricted eligibility to individuals aged 50 years or older and was closed once the target number of respondents was achieved in all strata.

### Participants (inclusion/exclusion criteria)

[First survey] We included participants aged 75 years and older who were relatively independent, defined by a care level of 2 or lower in the Level of Long-Term Care Insurance (LTCI) system [31, 32]. LTCI is a mandatory social insurance system, where a care level of 2 indicates that a person requires only limited assistance with basic activities of daily living, such as bathing, managing daily medications, or finances [31, 32]. This cutoff level has frequently been used in previous studies as a threshold for independent living [33–35]. We excluded residents who had been hospitalized for longer than six months or who had declined to participate in the study. Of the 10,233 citizens aged 75 or older in the city as of February 2022, we distributed 8,820 paper-based, self-administered questionnaires to eligible residents.

[Second survey] We included participants ≥ 50 years of age who could complete an internet survey. We planned in advance to exclude non-serious respondents using two distinctive attention check questions (Directed Questions Scale and Instrumental Manipulation Check) and manually reviewed responses for obvious inconsistencies to minimize response bias inherent in internet surveys [36, 37].

Since this research is part of a larger study project, we were unable to control the number of participants. However, we have confirmed that the sample size is sufficient to validate the BPS in adherence to the COSMIN guidelines [38].

### Validation of the JBPS among older adults

The original Boredom Proneness Scale (BPS), developed in 1986, was not constructed based on a predefined theoretical structure [18]. As such, its factorial validity has remained unclear, with prior studies reporting inconsistent factor solutions across different populations [23, 39–41]. Given this background, we treated the Japanese version of the BPS (JBPS) as a reflective scale and assessed its structural validity in an exploratory framework, following the COSMIN guidelines. We utilized data from the First Survey only to validate the JBPS, ensuring methodological independence from the Second Survey used for other purposes. In accordance with previous similar studies [39–41] the validation process comprised four main steps: First, we examined item-level statistics including mean, standard deviation, skewness, kurtosis, item difficulty, item discrimination, and Cronbach’s α if deleted. Reliability was assessed using internal consistency (Cronbach’s α, expected range: 0.70–0.95; mean inter-item correlation: 0.15–0.50), test-retest reliability (intraclass correlation coefficient [ICC] > 0.70, two-way random-effects, absolute-agreement, single-measurement), and measurement error (standard error of measurement (SEM) < 10% of score range; minimal detectable change < 30% of the score range, calculated as 1.96 × √2 × SEM corresponding to the 95% confidence level). Second, we randomly split the sample into two halves to conduct Exploratory Factor Analysis (EFA) and Confirmatory Factor Analysis (CFA) independently. EFA was performed using varimax rotation on one half (*n* = 1,866). To determine the number of underlying factors, we conducted Velicer’s Minimum Average Partial (MAP) test both before and after removing reverse-worded items (#1, #7, #8, #11, #13, #15, #18, #22, #23, #24), following a methodology similar to that of a previous study [40]. Third, confirmatory factor analysis (CFA) was conducted on the remaining half of the sample (*n* = 1,865) to evaluate 2- to 5-factor models informed by the EFA results. Third, confirmatory factor analysis (CFA) was conducted on the remaining half of the sample (*n* = 1,865) to evaluate 2- to 5-factor models informed by the EFA results. The CFA aimed to verify whether the factorial structures reported in previous studies—particularly the recent German validation by Zerr et al. [40] —could be replicated in our sample of older Japanese adults. Consistent with that study, CFAs were estimated using the robust maximum likelihood estimator (MLR), ensuring methodological comparability with prior literature. To assess robustness and evaluate the potential impact of missing data, we additionally performed multiple imputation (MI) using chained equations and re-estimated the CFAs with both MLR and the weighted least-squares mean and variance adjusted (WLSMV) estimator, which is theoretically more appropriate for ordinal data. Model fit was assessed using standard indices (GFI > 0.95, AGFI > 0.95, CFI > 0.90, TLI > 0.90, RMSEA < 0.06, SRMR < 0.08) [42–44]. Fourth, we assessed hypothesis testing for construct validity, in accordance with COSMIN terminology, which included both criterion and convergent validity. Criterion validity was evaluated by correlating JBPS scores with single-item self-ratings of boredom and interest from the original derivation study [18]. Convergent validity was examined via correlations with measures of physical and mental health (SF-8 PCS and MCS) [45], UCLA loneliness scale [46, 47], Kessler Psychological Distress Scale-6 (K6) [48], Geriatric Depression Scale-15(GDS-15) [49], and EuroQol 5 dimensions 5-level (Eq. 5D-5 L) [50, 51]. Based on prior theoretical and empirical work, we hypothesized that higher boredom proneness would be associated with greater psychological distress, depressive symptoms, and loneliness, as well as lower health-related quality of life and overall well-being. These expected associations are theoretically grounded in the concept that boredom proneness reflects diminished engagement with meaningful activities, leading to emotional dysregulation and social withdrawal. These indices have been validated among the Japanese population and are potentially correlated with boredom proneness among older populations. Additionally, we assessed monotonicity using Mokken analysis (item scalability coefficient H > 0.3) and local independence using Q3 residuals (mean residual < 0.2), in line with item response theory (IRT).

### Derivation and validation of the s-BPSO

We developed the short version of the Boredom Proneness Scale for Older adults (s-BPSO) in four steps. First, we evaluated the structural validity of 12 existing short BPS versions [39, 41] using CFA based on the First Survey data. None demonstrated adequate model fit in our older sample. Second, we developed a new short scale based on a two-factor structure, which has been frequently supported in major previous research [39], and aligns with the original BPS concept that boredom is the psychological opposite of interest [18]. Prior studies also indicate that reverse-worded items may load onto a separate factor [40, 41]. Therefore, we selected items separately from reverse- and non-reverse-worded pools using IRT with a graded response model [52], prioritizing discrimination, difficulty, monotonicity, and test information function (TIF), alongside internal consistency and model fit. Third, we validated the s-BPSO using data from the Second Survey, employing the same reliability and structural validity procedures described for the JBPS. Fourth, we conducted hypothesis testing for construct validity, following the same procedure used for the JBPS. Criterion validity was evaluated using single-item self-ratings of boredom and interest from the original BPS derivation study [18], and convergent validity was examined through correlations with relevant psychological and health measures. Furthermore, we illustrated the psychometric characteristics of the scale using item characteristic curves and test information functions.

### Additional description of the JBPS and s-BPSO

We visualized the distribution of JBPS and s-BPSO across sex, age, education status, annual household income, number of household members, status of IADL, and HRQOL in the first survey using histograms and kernel density estimation. This exploratory analysis was conducted to illustrate the overall distribution and crude associations between JBPS/s-BPSO scores and these fundamental variables, providing descriptive context for future research. The *t*-test was used to compare the mean differences of BPS across binary variables, and the Jonckheere-Terpstra trend test was used to assess the trend of non-binary variables. Statistical significance was determined as a 2-sided *p*-value < 0.05. We conducted the main analyses using a complete-case approach. All statistical analyses were conducted using R version 4.3.2 (R Foundation for Statistical Computing, Vienna, Austria). Details of the packages used are provided in Supplementary methods.

## Results

### Participants characteristics

**Fig. 1:**
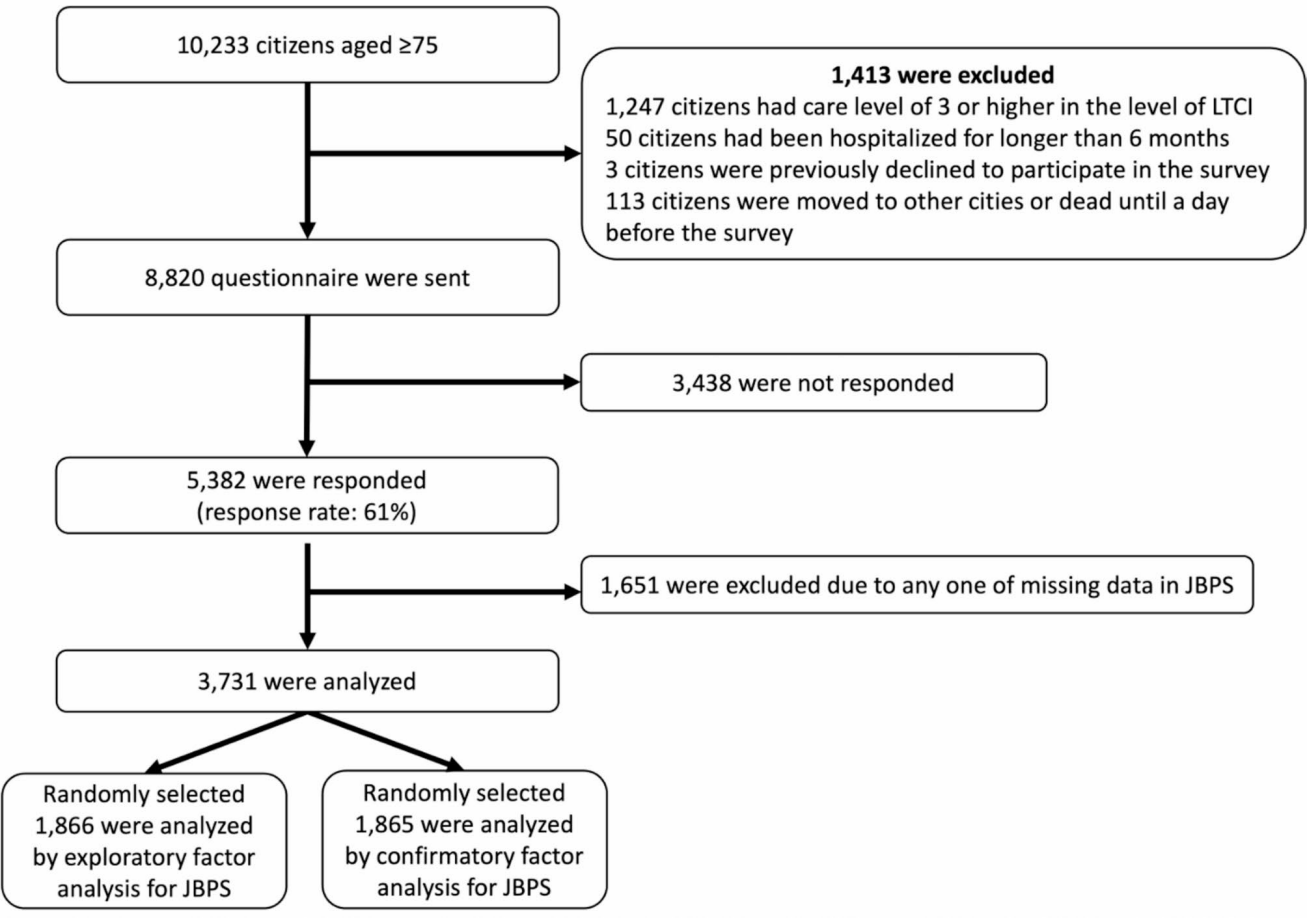
Study flow diagram (First survey). Notes, LTCI: Long-Term Care Insurance, JBPS: Japanese version of the Boredom Proneness Scale

In the first survey, we collected 5,382 responses (response rate: 61%, 5,382/8,820), of which 1,651 were excluded due to missing data on JBPS, as shown in Fig. 1. The remaining 3,731 participants were analyzed; 54.1% were women, with a mean age and standard deviation (SD) of 82 (4.72) years. The mean (SD) scores for JBPS and s-BPSO in the first survey were 97.40 (15.64) and 19.93 (6.32), respectively. In the second survey, after excluding 447 non-serious responders, 4,124 participants were analyzed; 54.0% were women, with a mean age of 66 (8.83) years. No missing data were reported for JBPS questions in the second survey due to the automated nature of internet surveys. The mean (SD) scores for JBPS and s-BPSO in the second survey were 96.70 (15.61) and 18.16 (5.87), respectively. The range of reference values (or 95% Confidence Interval) for BPS and s-BPSO among Japanese older adults were 66–128 and 9–33 in the first survey. Table 1 provides more detailed information on the participant characteristics of both surveys. To further clarify the age composition, we also summarized the characteristics of participants aged 75 years and older in each survey, as shown in Supplementary Table S1.

**Table 1:** Characteristics of the participants

	First survey		Second survey
	(*N* = 3,731)		(*N* = 4,124)
Sex (Men, %)	1,523 (45.9)		1,895 (46.0)
Age (year, Mean, SD)	82 (4.72)		66 (8.83)
Married (n, %)	3,674 (98.5)		2,979 (72.2)
Graduated from high school or upper level (n, %)	2,071 (55.5)		4,023 (97.6)
Educational background (n, %)			
Graduated from elementary school	111 (3.0)		0 (0.0)
Graduated from junior high school	1,549 (41.5)		90 (2.2)
Graduated from high school	1,352 (36.2)		1,515 (36.7)
Graduated from junior college	82 (2.2)		598 (14.5)
Graduated from professional training college	249 (6.7)		375 (9.1)
Graduated from university	238 (6.4)		1,422 (34.5)
Graduated from graduate school	10 (0.3)		113 (2.7)
Others	42 (1.1)		11 (0.3)
Missing	98 (2.7)		–
Number of Family members (n, %)			
Living alone	550 (14.7)		758 (18.4)
2 persons	1,427 (38.2)		2,065 (50.1)
3 persons	717 (19.2)		806 (19.5)
4 persons	413 (11.1)		363 (8.8)
5 persons or more	582 (15.6)		132 (3.2)
Missing	42 (1.2)		–
Caregiving (n,%)			
Highly committed	128 (3.4)		158 (3.8)
Partially committed	152 (4.1)		177 (4.3)
None	3,373 (90.4)		3,789 (91.9)
Missing	78 (2.1)		–
Smoking habits (n, %)			
Never	2,173 (58.2)		2,113 (51.2)
Past	1,228 (32.9)		1,367 (33.1)
Current	241 (6.5)		644 (15.6)
Missing	89 (2.4)		–
Drinking habit (n, %)			
Non	2,545 (68.2)		1,892 (45.9)
1–2 days/week	269 (7.2)		736 (17.8)
3–4 days/week	192 (5.1)		448 (10.9)
5–6 days/week	187 (5.0)		226 (5.5)
Everyday	470 (12.6)		223 (5.4)
Missing	68 (1.8)		–
Height, cm, (Mean, SD)	155.23 (9.08)		161.86 (9.01)
Weight, kg, (Mean, SD)	56.08 (10.08)		58.88 (12.55)
Past medical history (n, %)			
Malignant disease	676 (18.9)		402 (9.7)
Stroke	101 (2.9)		128 (3.1)
Myocardial infarction	411 (11.8)		143 (3.5)
Hypertension	2,141 (60)		1,372 (33.3)
Diabetes	609 (17.3)		438 (10.6)
Depression	69 (2.0)		203 (4.9)
Dementia	102 (2.9)		13 (0.3)
UCLA 3 Item Loneliness Scale (Mean, SD)	3.89 (1.44)		4.31 (1.55)
K6 (Mean, SD)	3.54 (4.03)		2.68 (3.82)
GDS15	–		3.99 (3.87)
Physical component score in SF-8 (Mean, SD)	46.4 (7.38)		–
Mental component score in SF-8 (Mean, SD)	50.28 (6.48)		–
Equation 5D-5 L	–		0.92 (0.11)
Annual household income (million JPY), (n, %)			
< 1.00 ($6,593 USD)	632 (16.9)	< 1.99 ($ 13,132 USD)	406 (10.5)
1.00–1.99	1,032 (27.7)	2.00–3.99	1,116 (28.9)
200–299	715 (19.2)	4.00–5.99	790 (20.4)
300–399	440 (11.8)	6.00–7.99	451 (11.7)
400–499	233 (6.2)	8.00–9.99	287 (7.4)
500–749	166 (4.4)	10.00–11.99	159 (4.1)
750–999	114 (3.1)	12.00–14.99	94 (2.4)
≥ 1,000	68 (1.8)	15.00–19.99	53 (1.4)
Missing	331 (8.9)	≥ 20.00	38 (1.0)
		Refused to answer	472 (12.2)

Notes, SD: standard deviation, K6: Kessler Psychological Distress Scale-6, GDS-15: Geriatric Depression Scale-15, SF-8: eight-item Short Form Health Survey, Eq. 5D-5 L: EuroQol 5 dimensions 5-level, JPY: Japanese Yen, USD: United States dollar

The exchange rate JPY/USD was used rate at March 2021

### Validation of JBPS among older population

The single-item statistics, item scalability coefficient, and Q3 residual statistics for the JBPS in the first survey are detailed in supplemental Table S2. The standard deviation ranged from a minimum of 1.06 to a maximum of 1.90, and item difficulty ranged from 0.26 to 0.62. The internal consistency of the JBPS sum score, measured by Cronbach’s α, was 0.868, and the mean inter-item correlation was 0.194. ICC, SEM, MDC were 0.84, 6.21 (3.3% of the possible score range), and 17.22 (9.1% of the possible score range). In the exploratory factor analysis of the JBPS, Velicer’s MAP test suggested a 3-factor structure. The loading patterns of single items on the three factors are shown in supplemental Table S3 (Total R2: 0.37, Factor 1: 0.173, Factor 2: 0.109, Factor 3: 0.089). After excluding the reverse-worded items, Velicer’s MAP test suggested a single-factor structure, aligning with Zerr’s validation results. The confirmatory factor analysis assuming a 3-factor structure indicated insufficient goodness-of-fit (GFI:0.859, AGFI: 0.833, CFI: 0.787, TLI: 0.768, RMSEA: 0.076, SRMR:0.082). Supplemental Tables S4-S7 show the loading patterns of single items on the 2-, 4-, and 5-factor models. The other confirmatory factor analyses for 2–5 factor structures also showed insufficient goodness-of-fit, but these findings are consistent with those in previous studies (Supplemental Table S6). Supplementary Table S8 summarizes the model fit indices obtained using the WLSMV estimator. The overall pattern was consistent with that obtained using MLR, with slightly higher comparative fit indices (CFI/TLI) under WLSMV. Additional CFAs conducted after multiple imputation (Supplementary Tables S9 and S10) yielded similar results, confirming the robustness of the factorial structure across estimation methods and missing-data handling.

The Spearman correlation coefficients between JBPS and various measures including self-ratings of boredom, interest, Physical Component Score (PCS) and Mental Component Score (MCS) in SF-8, UCLA loneliness scale, K6, GDS-15, Eq. 5D-5 L were 0.516, -0.224, -0.305, -0.368, 0.382, 0.516, 0.599, and − 0.323, respectively (Table 2). The directions of those correlations were reasonable along with previous studies and our priori hypotheses. Correlations of JBPS to GDS15 and Eq. 5D-5 L were derived from the second survey.

**Table 2:** Correlation of the Japanese version of boredom proneness scale to the potentially boredom-related indices

	Self-ratings of boredom	Self-ratings of interest	PCS in SF-8	MCS in SF-8	UCLA loneliness scale	K6	GDS-15	Equation 5D-5L
*N*	3,731	3,731	3,731	3,731	3,731	3,731	4,124	4,124
Spearman R	0.516	-0.224	-0.305	-0.368	0.382	0.516	0.599	-0.323
*P* value	< 0.001	< 0.001	< 0.001	< 0.001	< 0.001	< 0.001	< 0.001	< 0.001

Notes, K6: Kessler Psychological Distress Scale-6, GDS-15: Geriatric Depression Scale-15, PCS: Physical component score, MCS: Mental component score, SF-8: eight-item Short Form Health Survey, Eq. 5D-5 L: EuroQol 5 dimensions 5-level

Correlations to the Self-ratings of boredom, interest, PCS in SF-8, MCS in SF-8, UCLA lonliness scale, and K6 were derived from the first survey. Correlations to the GDS15 and Eq. 5D-5 L were derived from the second survey

### Derivation and validation of the s-BPSO

Due to the inadequate model fit of all existing short versions of the BPS for our older adult sample (Supplemental Table S11), we developed a new 6-item version (s-BPSO) based on a two-factor structure, selecting items separately from reverse- and non-reverse-worded pools using graded response IRT parameters (discrimination, difficulty, test information), internal consistency, and monotonicity. The selected items (#4, #14, #16; #7, #8, #13) demonstrated strong psychometric properties and conceptual relevance for assessing boredom proneness in this population. Supplemental Table S12 shows Single-item statistics and Item Scalability Coefficient and Q3 residual statistics. The mean inter-item correlation was 0.408, Cronbach’s α was 0.803, Omega Total was 0.84, and Omega Hierarchical was 0.65. ICC, SEM MDC were 0.83, 2.44 (6.97% of the possible score range), 6.77(19.34% of the possible score range). Supplemental Figure S1and S2 show item characteristics curves for each item and test information function of the s-BPSO. Confirmatory factor analysis assuming a two-factor model for the s-BPSO using the second survey was performed (Supplemental Figure S3) and indicated acceptable goodness-of-fit (GFI: 0.991, AGFI: 0.976, CFI: 0.987, TLI: 0.976, RMSEA: 0.056, SRMR: 0.022). A similar analysis using the second survey data from participants aged 75 years and older also showed acceptable goodness of fit (GFI: 0.985, AGFI: 0.961, CFI: 0.983, TLI: 0.968, RMSEA: 0.065, SRMR: 0.024). The Spearman correlation coefficients between the s-BPSO and JBPS, self-ratings of boredom, interest, UCLA loneliness scale, K6, GDS-15, and Eq. 5D-5 L were 0.857, 0.547, -0.557, 0.371, 0.414, 0.524, and − 0.273, respectively.

### JBPS and s-BPSO distribution across fundamental variables among older population

**Fig. 2:**
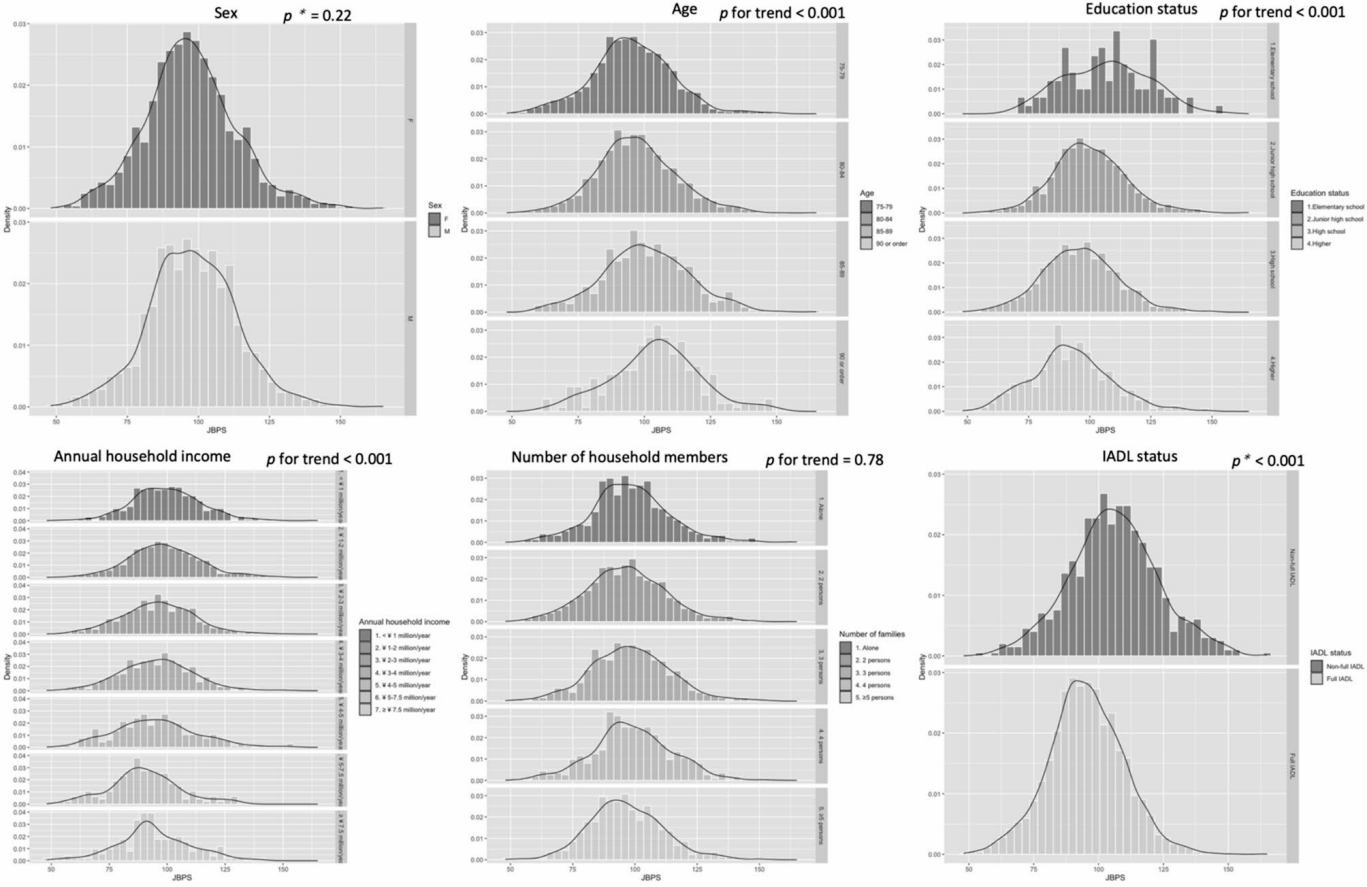
The distribution of JBPS across socioeconomic factors among community dwelling older population. Notes, p* values calculated using *t* test. The other *p* for trend values were calculated by Jonckheere-Terpstra trend test

**Fig. 3:**
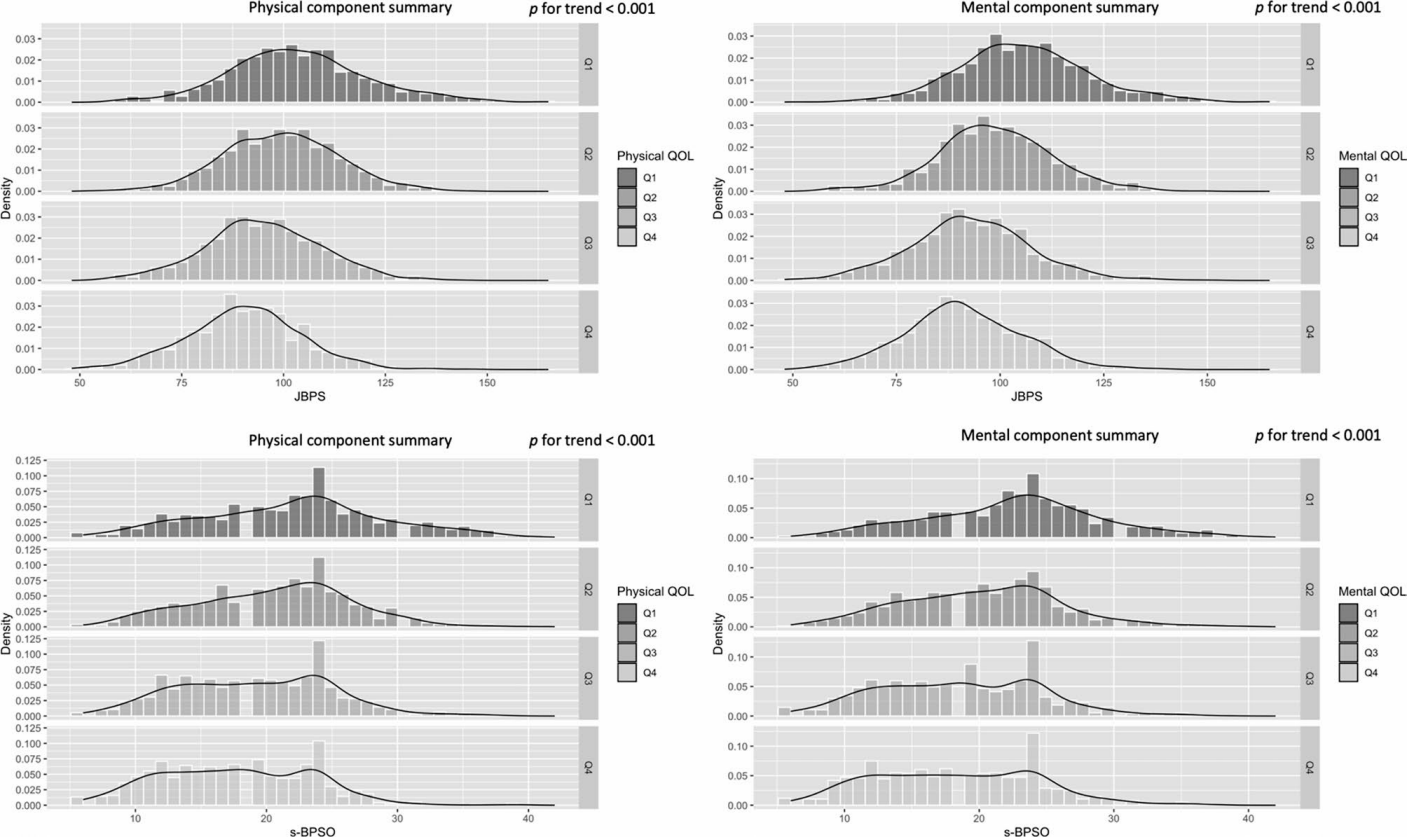
The distribution of JBPS and s-BPSO across HRQOL among community dwelling older population. Notes, *p* for trend values were calculated by Jonckheere-Terpstra trend test

Figure 2 illustrates the distribution of the JBPS across sex, age, education status, annual household income, number of household members, and status of IADL. It appears that older age, lower education, lower annual household income, and lack of full IADL are correlated with higher JBPS scores. Supplemental Figure S4 displays the distribution of the s-BPSO and indicates trends similar to those observed with the JBPS. Figure 3 demonstrates the distribution of JBPS and s-BPSO across the PCS and MCS in the SF-8. Both higher mental and physical component scores seem to be correlated with lower JBPS and s-BPSO scores in a dose-dependent manner.

## Discussion

### Key findings

In this study, we validated the JBPS, developed and validated the s-BPSO, and examined their utility. We found that the JBPS demonstrated a factor structure and correlations with other psychometric indices comparable to those of the original BPS version. Furthermore, our findings revealed that the existing short versions of the BPS did not satisfactorily fit the older Japanese population. Our newly developed s-BPSO demonstrated satisfactory reproducibility regarding internal consistency, test-retest reliability, and factor structure and correlations with other psychometric measures related to boredom proneness within a different sample of the older population. Consequently, we confirmed that both JBPS and s-BPSO are suitable for assessing boredom proneness among the older Japanese population.

### Comparison with previous studies

Numerous studies have highlighted boredom as a key factor influencing quality of life in later life [53, 54], given its significant associations with depression [5–8], anxiety [7, 9], loneliness [8, 11], and social isolation [12]. However, boredom proneness among older adults has rarely been examined using reliable measures in large samples [25]. Our study addresses this gap by characterizing boredom proneness in older Japanese adults and introducing a practical assessment tool. We described BPS distributions and proposed reference values. Boredom proneness was correlated with age, education, income, IADL status, and HRQOL. Previous studies on gender differences in BPS show inconsistent results, with some reporting higher scores in men [39, 55, 56], and others reporting no or opposite relations [57–60]. A research including adults up to age 63 also suggests boredom decreases with age [61]. While generalizability warrants further investigation, our findings offer an initial step toward understanding how boredom impacts older adults’ health and well-being.

In our two distinct surveys, the JBPS consistently demonstrated sufficient reliability, and its structural, criterion-related, and convergent validity were confirmed. However, a previous study from France reported findings that did not align with ours [62]. Gana et al. conducted a longitudinal assessment of the BPS among community-living older adults (mean age 72.50 years, SD 5.89) over a six-year period to determine whether the BPS measures inherent boredom proneness or is influenced by the participants’ transient states [62]. Despite the lower reliability of a binary response format (true-false) compared to the 7-point Likert scale [24], they employed a binary response. They concluded with skepticism regarding the BPS’s utility. However, their reported reliability indices were far below the acceptable threshold in the first place (specific reasons were not articulated in the manuscript), a finding at odds with numerous preceding studies [40, 41, 63, 64]. There are inherent differences between their findings and ours; drawing any conclusions from an unreliable measure is inherently problematic.

Although many previous studies have not reached a consensus on the number of factor structures of the BPS in confirmatory factor analysis [24], our exploratory and confirmatory factor analyses are highly consistent with the results of a recent validation study in Germany [40]. The inconsistent results among previous studies regarding the number of factor structures are often explained by differences in the measurement scale (true-false / 7-point Likert scale), cutoff reference of factor loadings, rotation method, and the characteristics of the targeted population [23, 24]. Thus, we selected their study as a reference because the characteristics of the population are sufficiently comparable to our own, and we strictly followed their validation process. Their results of exploratory factor analysis, exploratory factor analysis after excluding the reverse-worded items, and the subsequent confirmatory factor analyses are all highly consistent with our results. Simultaneously, the JBPS also indicated a fairly strong correlation with measures of depression, loneliness psychometrics, and HRQOL, in line with previous studies. Therefore, these results indicate the acceptable validity of the JBPS in accordance with the previous validation processes [40, 63].

### Limitations

First, we validated the s-BPSO only in a Japanese population; its applicability to other nationalities remains untested and requires further validation. Second, although the s-BPSO was derived from JBPS item responses within the same dataset, the short version itself has not yet been validated as a standalone instrument. Because the present study did not distribute or evaluate the s-BPSO independently from the JBPS, its external validity and measurement equivalence when used alone remain to be established. Further independent validation studies are warranted. Third, we did not evaluate responsiveness; thus, the ability of the JBPS and s-BPSO to detect changes over time remains unknown. Fourth, due to the cross-sectional design, causal relationships cannot be inferred, and unadjusted correlations may be confounded. Fifth, factors such as national character [65], other psychological features [66], and historical backgrounds [67], which may influence boredom proneness, were not assessed. Sixth, in the first survey, participants were drawn from the Late Elders’ Health Insurance registry, which excludes welfare recipients, potentially omitting socially disadvantaged individuals. Seventh, in the second survey, only internet users with adequate digital literacy could participate, introducing possible selection bias. Eighth, although no missing data were recorded for JBPS or s-BPSO items in the second survey due to the automated response system, this does not preclude potential bias from selective participation. Individuals who were unwilling to respond to certain items might have either discontinued the survey before completion or decided not to participate at all, which could have led to the underrepresentation of such participants. Ninth, some JBPS items showed low discrimination, difficulty, monotonicity, and information in IRT analysis, suggesting limited measurement precision. The 7-point Likert scale may also reduce scalability due to response burden or interpretability issues [68]. Further research is warranted to explore and address these limitations. Finally, although the present study represents a necessary first step in investigating boredom among Japanese older adults, the original BPS was developed nearly four decades ago and was not specifically designed for older populations or based on contemporary theoretical frameworks for patient-reported outcome measures. Moreover, its item content may not fully reflect the sociocultural and psychological context of modern aging. Therefore, while the current validation provides an important empirical foundation, future research should aim to reconceptualize boredom in alignment with current psychosocial theories and develop refined measurement tools tailored to the purposes and experiences of today’s older adults.

## Conclusion

In summary, the present study demonstrated that both the JBPS and s-BPSO exhibit acceptable psychometric properties for assessing boredom proneness among older Japanese adults. Internal consistency and test–retest reliability were within acceptable ranges, indicating stable and coherent measurement. Structural validity was supported through exploratory and confirmatory factor analyses, which replicated previously reported factorial patterns. Furthermore, hypothesis testing for construct validity revealed theoretically consistent associations with measures of mental health, loneliness, and quality of life. Together, these findings support the reliability and validity of both the JBPS and s-BPSO as tools for evaluating boredom proneness in this population, although further studies using independent samples are warranted to confirm the external validity of the s-BPSO when administered as a standalone instrument.

## Supplementary Information

Below is the link to the electronic supplementary material.

Supplementary Material 1

## Data Availability

The datasets used and analyzed during the study are available from the corresponding author upon request and subject to ethical approval.

## Abbreviations

JBPS: Japanese version of the Boredom Proneness Scale
s-BPSO: Short version of the Boredom Proneness Scale for older people
IADL: Instrumental Activity of Daily Living
IRT: Item Response Theory
HRQOL: Health-Related Quality of Life
