# Supplementary material for: Validation of the Japanese version of boredom proneness scale and derivation of its short version among older adults: a cross-sectional study
Source: Health Qual Life Outcomes. 2026 Feb 4;24:31. doi: 10.1186/s12955-026-02490-3 (PMC12958760; doi:10.1186/s12955-026-02490-3)
Supplement: Supplementary file 1 — Supplementary Material 1 [file 12955_2026_2490_MOESM1_ESM.docx]

**Supplemental materials**

Title

Validation of the Japanese version of the Boredom Proneness Scale and derivation of its short version for older adults: A Cross-Sectional Study

CONTENTS

**Supplemental methods**

- Details of the questionnaires used in this study
- List of R packages used in this study

**Supplemental tables & figures**

- **Supplementary Table S1.** Participant characteristics of individuals aged 75 years and older in the first and second surveys.
- **Supplementary Table S2** Single item statistics of the JBPS in the first survey (N = 3,731)
- **Supplementary Table S3**. Exploratory factor analysis of the JBPS, loadings on a suggested 3-factor structure (N = 1,866)
- **Supplementary Table S4.** Experimental exploratory factor analysis of the JBPS, loadings on a 2-factor structure (N = 1,865)
- **Supplementary Table S5.** Experimental exploratory factor analysis of the JBPS, loadings on a 4-factor structure (N = 1,865)
- **Supplementary Table S6.** Experimental exploratory factor analysis of the JBPS, loadings on a 5-factor structure (N = 1,865)
- **Supplementary Table S7.** Model goodness-of-fit indices from confirmatory factor analyses (CFA) of the JBPS under 2- to 5-factor models estimated using robust maximum likelihood (MLR)
- **Supplementary Table S8.** Model goodness-of-fit indices from confirmatory factor analyses (CFA) of the JBPS under 2- to 5-factor models estimated using weighted least-squares mean and variance adjusted (WLSMV)
- **Supplementary Table S9.** Model goodness-of-fit indices from confirmatory factor analyses (CFA) of the JBPS under 2- to 5-factor models estimated using robust maximum likelihood (MLR) after multiple imputation
- **Supplementary Table S10.** Model goodness-of-fit indices from confirmatory factor analyses (CFA) of the JBPS under 2- to 5-factor models estimated using weighted least-squares mean and variance adjusted (WLSMV) after multiple imputation
- **Supplementary Table S11.** Model goodness-of-fit in the confirmatory factor analysis for previous reported short versions of BPS
- **Supplementary Table S12.** Single item statistics of the s-BPSO in the second survey
- **Supplementary Figure S1.** Item Characteristics Curve for each item in s-BPSO
- **Supplementary Figure S2.** Test Information Function Plot of s-BPSO
- **Supplementary Figure S3.** Structural diagram of the two-factor model for the s-BPSO using the second survey
- **Supplementary Figure S4.** The distribution of s-BPSO across socioeconomic factors among community-dwelling older population

**Supplemental methods**

***Details of original questionnaires used in this study***

(We also used Japanese version of Boredom Proneness Scale, SF-8, UCLA loneliness scale, K-6, GDS-15, and EQ5D-5L.)

*Height*

How tall are you?

____cm

*Weight*

How much do you weigh?

_____kg

*Past medical history*

Please choose diseases which you have been diagnosed with.

Cancer

Stroke

Acute myocardial infarction/ Angina pectoris

Hypertension

Diabetes

Depression

Dementia

*Smoking habits*

Do you smoke?

1. Never smoke.

2. I used to smoke.

3. I currently smoke.

*Drinking habits*

How often do you drink alcohol of more than 20g ethanol (500 ml beer, a cup of Japanese sake, or 100 ml Japanese shochu) in a week?

0. Never

1. 1 to 2 times a week

2. 3 to 4 times a week

3. 5 to 6 times a week

4. Everyday

*Instrumental Activity of Daily Living (IADL)*

Do you independently travel on public transportation such as bus or train?

Please choose the one which applies to your current situation.

1. Yes

2. If someone assists me.

3. No

Do you go shopping to buy daily necessities?

Please choose the one which applies to your current situation.

1. Yes

2. If someone assists me.

3. No

Do you independently prepare meal?

Please choose the one which applies to your current situation.

1. Yes

2. If someone assists me.

3. No

Do you independently pay your bills?

Please choose the one which applies to your current situation.

1. Yes

2. If someone assists me.

3. No

Do you independently deposit and withdraw money from the bank?

Please choose the one which applies to your current situation.

1. Yes

2. If someone assists me.

3. No

*Number of Family members*

How many people do you live together aside from you?

1. none (alone)

2. one

3. two

4. three

5. four or more

*Caregiving*

Are you giving care to someone in your family?

1. Yes. I am the main caregiver for the person.

2. Yes. I partially support the person, not as a main caregiver.

3. No.

*Educational background*

Please choose the highest education you have graduated from.

1. Elementary school

2. Junior high school

3. High school

4. Junior college

5. Professional training college

6. University

7. Graduate school

8. Others

*Annual household income*

What was your household's annual income (including pension and before tax deduction) in the last year? Please choose from the following:

1. JPY <1,000,000

2. JPY 1,000,000–1,999,999

3. JPY 2,000,000–2,999,999

4. JPY 3,000,000–3,999,999

5. JPY 4,000,000–4,999,999

6. JPY 5,000,000–7,499,999

7. JPY 7,500,000–10,000,000

8. JPY ≥10,000,000

*Marital status*

Please choose your marital status from the following.

1. Never married

2. Married and living together

3. Married but not living together

4. Separated

5. Widowed

6. Others

*Self-ratings boredom*

How often do your feel bored?

1. Almost always

2. Often

3. Sometimes

4. Seldom

5. Never

*Self-ratings interest*

How much of the time are you satisfied or interested in what you are doing?

1. Almost always

2. Often

3. Sometimes

4. Seldom

5. Never

***Attention check question***

Please choose “strongly agree” from the bottom of the following options.

1. strongly agree

2. agree

3. slightly agree

4. neutral

5. slightly disagree

6.disagree

7. strongly disagree

Please neglect the following question regarding electronic devices and choose “others” from the bottom of the following options.

Which electronic devices do you usually use most often?

1. laptop personal computer

2. desktop personal computer

3. television

4. smartphone

5. radio

6. others

**List of R packages used in this study**

All analyses were conducted using R version 4.3.2 (R Foundation for Statistical Computing, Vienna, Austria). The main packages employed were: lavaan (for confirmatory factor analysis), psych (for exploratory factor analysis), semTools (for structural validity and reliability indices), mokken (for Mokken scale analysis), ltm and mirt (for item response theory analyses), irr (for intraclass correlation coefficients), effectsize (for effect size estimation), and tidyverse and dplyr (for data management and visualization).

**Supplementary Table S1.** Participant characteristics of individuals aged 75 years and older in the first and second surveys.

|  | **First survey** |  | **Second survey**  **(participants aged ≥75 years)** |
| --- | --- | --- | --- |
|  | **(N=3,731)** |  | **(N=725)** |
| Sex (Men, %) | 1,523 (45.9) |  | 305 (42.1) |
| Age (year, Mean, SD) | 82 (4.72) |  | 77 (2.56) |
| Married (n, %) | 3,674 (98.5) |  | 519 (71.6) |
| Graduated from high school or upper level (n, %) | 2,071 (55.5) |  | 698 (96.3) |
| Educational background (n, %) |  |  |  |
| Graduated from elementary school | 111 (3.0) |  | 0 (0.0) |
| Graduated from junior high school | 1,549 (41.5) |  | 27 (3.7) |
| Graduated from high school | 1,352 (36.2) |  | 319 (44.0) |
| Graduated from junior college | 82 (2.2) |  | 88 (12.2) |
| Graduated from professional training college | 249 (6.7) |  | 44 (6.1) |
| Graduated from university | 238 (6.4) |  | 231 (31.9) |
| Graduated from graduate school | 10 (0.3) |  | 16 (2.2) |
| Others | 42 (1.1) |  | 0 (0.0) |
| Missing | 98 (2.7) |  | – |
| Number of Family members (n, %) |  |  |  |
| Living alone | 550 (14.7) |  | 141 (19.4) |
| 2 persons | 1,427 (38.2) |  | 440 (60.7) |
| 3 persons | 717 (19.2) |  | 103 (14.2) |
| 4 persons | 413 (11.1) |  | 22 (3.0) |
| 5 persons or more | 582 (15.6) |  | 19 (2.6) |
| Missing | 42 (1.2) |  | – |
| Caregiving (n,%) |  |  |  |
| Highly committed | 128 (3.4) |  | 20 (2.8) |
| Partially committed | 152 (4.1) |  | 17 (2.3) |
| None | 3,373 (90.4) |  | 688 (94.9) |
| Missing | 78 (2.1) |  | – |
| Smoking habits (n, %) |  |  |  |
| Never | 2,173 (58.2) |  | 408 (56.3) |
| Past | 1,228 (32.9) |  | 266 (36.7) |
| Current | 241 (6.5) |  | 51 (7.0) |
| Missing | 89 (2.4) |  | – |
| Drinking habit (n, %) |  |  |  |
| Non | 2,545 (68.2) |  | 361 (49.8) |
| 1 -2 days/week | 269 (7.2) |  | 126 (17.4) |
| 3 - 4 days/week | 192 (5.1) |  | 68 (9.4) |
| 5 - 6 days/week | 187 (5.0) |  | 38 (5.2) |
| Everyday | 470 (12.6) |  | 98 (13.5) |
| Missing | 68 (1.8) |  | – |
| Height, cm, (Mean, SD) | 155.23 (9.08) |  | 158.62 (9.80) |
| Weight, kg, (Mean, SD) | 56.08 (10.08) |  | 56.29 (11.53) |
| Past medical history (n, %) |  |  |  |
| Malignant disease | 676 (18.9) |  | 113 (15.6) |
| Stroke | 101 (2.9) |  | 28 (3.9) |
| Myocardial infarction | 411 (11.8) |  | 46 (6.3) |
| Hypertension | 2,141 (60) |  | 340 (46.9) |
| Diabetes | 609 (17.3) |  | 100 (13.8) |
| Depression | 69 (2.0) |  | 13 (1.8) |
| Dementia | 102 (2.9) |  | 6 (0.8) |
| UCLA 3 Item Loneliness Scale (Mean, SD) | 3.89 (1.44) |  | 3.84 (1.18) |
| K6 (Mean, SD) | 3.54 (4.03) |  | 1.64 (2.45) |
| GDS15 | – |  | 2.95 (3.27) |
| Physical component score in SF-8 (Mean, SD) | 46.4 (7.38) |  | – |
| Mental component score in SF-8 (Mean, SD) | 50.28 (6.48) |  | – |
| EQ5D-5L | – |  | 0.91 (0.12) |
| Annual household income (million JPY), (n, %) |  |  |  |
| < 1.00 ($6,593 USD) | 632 (16.9) | < 1.99 ($ 13,132 USD) | 78 (11.1) |
| 1.00 - 1.99 | 1,032 (27.7) | 2.00 - 3.99 | 290 (41.4) |
| 200 - 299 | 715 (19.2) | 4.00 - 5.99 | 143 (20.4) |
| 300 - 399 | 440 (11.8) | 6.00 - 7.99 | 61 (8.7) |
| 400 - 499 | 233 (6.2) | 8.00 - 9.99 | 29 (4.1) |
| 500 - 749 | 166 (4.4) | 10.00 - 11.99 | 16 (2.3) |
| 750 - 999 | 114 (3.1) | 12.00 - 14.99 | 11 (1.6) |
| ≥ 1,000 | 68 (1.8) | 15.00 - 19.99 | 5 (0.7) |
| Missing | 331 (8.9) | ≥ 20.00 | 3 (0.4) |
|  |  | Refused to answer | 65 (9.3) |

**Notes**, SD: standard deviation, K6: Kessler Psychological Distress Scale-6, GDS-15: Geriatric Depression Scale-15, SF-8: eight-item Short Form Health Survey, EQ5D-5L: EuroQol 5 dimensions 5-level, JPY: Japanese Yen , USD: United States dollar

The exchange rate JPY/USD was used rate at March 2021.

**Supplementary Table S2 Single item statistics of the JBPS in the first survey (N = 3,731)**

| Items | Mean | SD | Skew | Kurtosis | Item Difficulty | Item Discrimination | α if deleted | Item Scalability Coefficient (H) |
| --- | --- | --- | --- | --- | --- | --- | --- | --- |
| bps1 | 3.76 | 1.90 | 0.00 | -1.40 | 0.54 | 0.31 | 0.87 | 0.19 |
| bps2 | 3.21 | 1.69 | 0.25 | -1.20 | 0.46 | 0.46 | 0.86 | 0.26 |
| bps3 | 2.94 | 1.62 | 0.68 | -0.60 | 0.42 | 0.37 | 0.86 | 0.24 |
| bps4 | 2.57 | 1.58 | 0.92 | -0.20 | 0.37 | 0.47 | 0.86 | 0.37 |
| bps5 | 2.19 | 1.32 | 1.34 | 1.44 | 0.31 | 0.47 | 0.86 | 0.36 |
| bps6 | 2.68 | 1.49 | 0.88 | 0.08 | 0.38 | 0.46 | 0.86 | 0.30 |
| bps7 | 3.86 | 1.89 | -0.07 | -1.34 | 0.55 | 0.41 | 0.86 | 0.37 |
| bps8 | 4.37 | 1.77 | -0.52 | -0.96 | 0.62 | 0.39 | 0.86 | 0.39 |
| bps9 | 3.48 | 1.67 | 0.21 | -1.11 | 0.50 | 0.52 | 0.86 | 0.31 |
| bps10 | 2.96 | 1.55 | 0.70 | -0.38 | 0.42 | 0.56 | 0.86 | 0.33 |
| bps11 | 3.08 | 1.56 | 0.53 | -0.61 | 0.44 | 0.47 | 0.86 | 0.34 |
| bps12 | 3.41 | 1.71 | 0.30 | -1.05 | 0.49 | 0.38 | 0.86 | 0.23 |
| bps13 | 4.25 | 1.71 | -0.37 | -0.98 | 0.61 | 0.34 | 0.87 | 0.41 |
| bps14 | 2.93 | 1.68 | 0.69 | -0.63 | 0.42 | 0.37 | 0.86 | 0.32 |
| bps15 | 3.94 | 1.67 | -0.09 | -0.99 | 0.56 | 0.34 | 0.87 | 0.29 |
| bps16 | 2.91 | 1.69 | 0.69 | -0.63 | 0.42 | 0.40 | 0.86 | 0.33 |
| bps17 | 3.05 | 1.66 | 0.65 | -0.61 | 0.44 | 0.40 | 0.86 | 0.27 |
| bps18 | 2.94 | 1.55 | 0.58 | -0.60 | 0.42 | 0.37 | 0.86 | 0.31 |
| bps19 | 3.94 | 1.79 | -0.04 | -1.18 | 0.56 | 0.36 | 0.86 | 0.27 |
| bps20 | 3.25 | 1.61 | 0.41 | -0.83 | 0.46 | 0.41 | 0.86 | 0.12 |
| bps21 | 3.08 | 1.52 | 0.58 | -0.53 | 0.44 | 0.48 | 0.86 | 0.30 |
| bps22 | 2.70 | 1.39 | 0.73 | -0.05 | 0.39 | 0.40 | 0.86 | 0.33 |
| bps23 | 2.94 | 1.56 | 0.74 | -0.28 | 0.42 | 0.36 | 0.86 | 0.35 |
| bps24 | 3.24 | 1.66 | 0.47 | -0.78 | 0.46 | 0.37 | 0.86 | 0.34 |
| bps25 | 1.85 | 1.06 | 1.70 | 3.57 | 0.26 | 0.38 | 0.86 | 0.23 |
| bps26 | 2.72 | 1.52 | 0.85 | -0.10 | 0.39 | 0.45 | 0.86 | 0.22 |
| bps27 | 3.11 | 1.60 | 0.57 | -0.69 | 0.44 | 0.44 | 0.86 | 0.27 |
| bps28 | 2.23 | 1.26 | 1.30 | 1.58 | 0.32 | 0.35 | 0.86 | 0.26 |

Mean inter-item-correlation=0.194, Cronbach's α=0.868

Q3 residual statistics (Min: -0.292, First quartile: -0.082, Median: -0.022, Mean: -0.021, Third quartile: 0.032, Max: 0.39)

Reference range of acceptable score (Mean inter-item-correlation 0.15–0.50, Cronbach’s α 0.70–0.95, Item scalability coefficients > 0.3, mean values of Q3 residuals < 0.2)

**Supplementary Table S3. Exploratory factor analysis of the JBPS, loadings on a suggested 3-factor structure (N = 1,866)**

| Items | Factor1 | Factor2 | Factor3 |
| --- | --- | --- | --- |
| bps1 |  |  | 0.366 |
| bps2 | 0.442 |  |  |
| bps3 | 0.435 |  |  |
| bps4 | 0.766 |  |  |
| bps5 | 0.685 |  |  |
| bps6 | 0.552 |  |  |
| bps7 |  |  | 0.597 |
| bps8 |  |  | 0.74 |
| bps9 | 0.532 |  | 0.322 |
| bps10 | 0.563 |  |  |
| bps11 |  | 0.446 | 0.388 |
| bps12 | 0.409 |  |  |
| bps13 |  | 0.312 | 0.673 |
| bps14 | 0.702 |  |  |
| bps15 |  |  | 0.47 |
| bps16 | 0.714 |  |  |
| bps17 | 0.456 |  |  |
| bps18 |  | 0.555 |  |
| bps19 | 0.494 |  |  |
| bps20 |  | 0.568 |  |
| bps21 | 0.491 |  |  |
| bps22 |  | 0.627 |  |
| bps23 |  | 0.646 |  |
| bps24 |  | 0.529 |  |
| bps25 | 0.316 | 0.517 |  |
| bps26 |  | 0.534 |  |
| bps27 | 0.425 |  |  |
| bps28 | 0.429 |  |  |

Total R^2^: 0.37, Factor 1: 0.173, Factor 2: 0.109, Factor 3: 0.089

Notes, JBPS: Japanese version of the Boredom Proneness Scale, bps: Boredom Proneness Scale, Cut off loading level < 0.3

**Supplementary Table S4. Experimental exploratory factor analysis of the JBPS, loadings on a 2-factor structure (N = 1,865)**

| Items | Factor1 | Factor2 |
| --- | --- | --- |
| bps1 |  |  |
| bps2 | 0.453 |  |
| bps3 | 0.439 |  |
| bps4 | 0.768 |  |
| bps5 | 0.693 |  |
| bps6 | 0.56 |  |
| bps7 |  | 0.577 |
| bps8 |  | 0.587 |
| bps9 | 0.529 |  |
| bps10 | 0.576 |  |
| bps11 |  | 0.587 |
| bps12 | 0.401 |  |
| bps13 |  | 0.662 |
| bps14 | 0.691 |  |
| bps15 |  | 0.438 |
| bps16 | 0.706 |  |
| bps17 | 0.466 |  |
| bps18 |  | 0.569 |
| bps19 | 0.483 |  |
| bps20 |  | 0.579 |
| bps21 | 0.506 |  |
| bps22 |  | 0.576 |
| bps23 |  | 0.651 |
| bps24 |  | 0.594 |
| bps25 | 0.343 |  |
| bps26 | 0.306 | 0.411 |
| bps27 | 0.44 |  |
| bps28 | 0.44 |  |

Total R2: 0.322, Factor 1: 0.177, Factor 2: 0.145

Notes, JBPS: Japanese version of the Boredom Proneness Scale, bps: Boredom Proneness Scale, Cut off loading level < 0.3

**Supplementary Table S5. Experimental exploratory factor analysis of the JBPS, loadings on a 4-factor structure (N = 1,865)**

| Items | Factor1 | Factor2 | Factor3 | Factor4 |
| --- | --- | --- | --- | --- |
| bps1 |  |  |  | 0.38 |
| bps2 |  | 0.498 |  |  |
| bps3 |  | 0.416 |  |  |
| bps4 |  | 0.683 | 0.411 |  |
| bps5 |  | 0.735 |  |  |
| bps6 |  | 0.559 |  |  |
| bps7 |  |  |  | 0.61 |
| bps8 |  |  |  | 0.736 |
| bps9 |  | 0.369 | 0.37 | 0.309 |
| bps10 |  | 0.423 | 0.355 |  |
| bps11 | 0.445 |  |  | 0.385 |
| bps12 |  |  | 0.355 |  |
| bps13 | 0.317 |  |  | 0.668 |
| bps14 |  | 0.316 | 0.714 |  |
| bps15 |  |  |  | 0.461 |
| bps16 |  | 0.306 | 0.746 |  |
| bps17 |  | 0.338 |  |  |
| bps18 | 0.545 |  |  |  |
| bps19 |  |  | 0.459 |  |
| bps20 | 0.586 |  |  |  |
| bps21 |  |  | 0.405 |  |
| bps22 | 0.626 |  |  |  |
| bps23 | 0.635 |  |  |  |
| bps24 | 0.529 |  |  |  |
| bps25 | 0.517 |  |  |  |
| bps26 | 0.555 |  |  |  |
| bps27 |  |  |  |  |
| bps28 |  | 0.314 |  |  |

Total R2: 0.392, Factor 1: 0.109, Factor 2: 0.105, Factor 3: 0.090, Factor 4: 0.088

Notes, JBPS: Japanese version of the Boredom Proneness Scale, bps: Boredom Proneness Scale, Cut off loading level < 0.3

**Supplementary Table S6. Experimental exploratory factor analysis of the JBPS, loadings on a 5-factor structure (N = 1,865)**

| Items | Factor1 | Factor2 | Factor3 | Factor4 | Factor5 |
| --- | --- | --- | --- | --- | --- |
| bps1 |  |  |  | 0.371 |  |
| bps2 |  | 0.44 |  |  |  |
| bps3 |  | 0.407 |  |  |  |
| bps4 |  | 0.713 | 0.308 |  |  |
| bps5 |  | 0.757 |  |  |  |
| bps6 |  | 0.513 | 0.31 |  |  |
| bps7 |  |  |  | 0.597 |  |
| bps8 |  |  |  | 0.722 |  |
| bps9 |  |  | 0.53 |  |  |
| bps10 |  | 0.354 | 0.475 |  |  |
| bps11 | 0.446 |  |  | 0.384 |  |
| bps12 |  |  | 0.42 |  |  |
| bps13 | 0.323 |  |  | 0.681 |  |
| bps14 |  | 0.347 | 0.445 |  | 0.556 |
| bps15 |  |  |  | 0.482 |  |
| bps16 |  | 0.331 | 0.468 |  | 0.604 |
| bps17 |  |  | 0.4 |  |  |
| bps18 | 0.55 |  |  |  |  |
| bps19 |  |  | 0.55 |  |  |
| bps20 | 0.585 |  |  |  |  |
| bps21 |  |  | 0.428 |  |  |
| bps22 | 0.633 |  |  |  |  |
| bps23 | 0.637 |  |  |  |  |
| bps24 | 0.529 |  |  |  |  |
| bps25 | 0.515 |  |  |  |  |
| bps26 | 0.544 |  |  |  |  |
| bps27 |  |  | 0.431 |  |  |
| bps28 |  |  | 0.35 |  |  |

Total R2: 0.410, Factor 1: 0.108, Factor 2: 0.092, Factor 3: 0.091, Factor 4: 0.086, Factor 5: 0.032

Notes, JBPS: Japanese version of the Boredom Proneness Scale, bps: Boredom Proneness Scale, Cut off loading level < 0.3

**Supplementary Table S7. Model goodness-of-fit indices from confirmatory factor analyses (CFA) of the JBPS under 2- to 5-factor models estimated using robust maximum likelihood (MLR)**

|  | GFI | AGFI | CFI | TLI | RMSEA | SRMR |
| --- | --- | --- | --- | --- | --- | --- |
| JBPS 2-factor model | 0.824 | 0.794 | 0.746 | 0.724 | 0.082 | 0.085 |
| JBPS 3-factor model | 0.859 | 0.833 | 0.787 | 0.768 | 0.076 | 0.082 |
| JBPS 4-factor model | 0.868 | 0.843 | 0.800 | 0.779 | 0.074 | 0.085 |
| JBPS 5-factor model | 0.894 | 0.873 | 0.838 | 0.820 | 0.065 | 0.072 |

**Caption:**
Model fit indices (GFI, AGFI, CFI, TLI, RMSEA, SRMR) for the 2- to 5-factor models of the Japanese version of the Boredom Proneness Scale (JBPS) estimated with the robust maximum likelihood estimator (MLR).
Reference values for acceptable fit are shown in parentheses (GFI ≥ 0.95, AGFI ≥ 0.95, CFI ≥ 0.90, TLI ≥ 0.90, RMSEA ≤ 0.06, SRMR ≤ 0.08).

**Supplementary Table S8. Model goodness-of-fit indices from confirmatory factor analyses (CFA) of the JBPS under 2- to 5-factor models estimated using weighted least-squares mean and variance adjusted (WLSMV)**

|  | GFI | AGFI | CFI | TLI | RMSEA | SRMR |
| --- | --- | --- | --- | --- | --- | --- |
| JBPS 2-factor model | 0.946 | 0.915 | 0.895 | 0.886 | 0.129 | 0.097 |
| JBPS 3-factor model | 0.951 | 0.923 | 0.904 | 0.896 | 0.120 | 0.093 |
| JBPS 4-factor model | 0.956 | 0.928 | 0.912 | 0.903 | 0.117 | 0.091 |
| JBPS 5-factor model | 0.966 | 0.946 | 0.934 | 0.927 | 0.101 | 0.080 |

**Caption:**
Model fit indices (GFI, AGFI, CFI, TLI, RMSEA, SRMR) for the 2- to 5-factor models of the Japanese version of the Boredom Proneness Scale (JBPS) estimated with the weighted least-squares mean and variance adjusted (WLSMV) estimator.
Comparative fit indices (CFI/TLI) were generally higher under WLSMV estimation compared with MLR, while the overall factorial structure remained consistent across estimators.

**Supplementary Table S9.** Model goodness-of-fit indices from confirmatory factor analyses (CFA) of the JBPS under 2- to 5-factor models estimated using robust maximum likelihood (MLR) after multiple imputation

|  | GFI | AGFI | CFI | TLI | RMSEA | SRMR |
| --- | --- | --- | --- | --- | --- | --- |
| JBPS 2-factor model | **–** | **–** | 0.733 | 0.711 | 0.081 | 0.086 |
| JBPS 3-factor model | **–** | **–** | 0.776 | 0.756 | 0.075 | 0.083 |
| JBPS 4-factor model | **–** | **–** | 0.748 | 0.723 | 0.079 | 0.109 |
| JBPS 5-factor model | **–** | **–** | 0.802 | 0.780 | 0.071 | 0.091 |

**Caption:**
Model fit indices (CFI, TLI, RMSEA, SRMR) for the 2- to 5-factor models of the Japanese version of the Boredom Proneness Scale (JBPS) estimated with the **robust maximum likelihood (MLR)** estimator after multiple imputation.
Reference values for acceptable fit are shown in parentheses: CFI ≥ 0.90, TLI ≥ 0.90, RMSEA ≤ 0.06, SRMR ≤ 0.08.

**Supplementary Table S10.** Model goodness-of-fit indices from confirmatory factor analyses (CFA) of the JBPS under 2- to 5-factor models estimated using weighted least-squares mean and variance adjusted (WLSMV) after multiple imputation

|  | GFI | AGFI | CFI | TLI | RMSEA | SRMR |
| --- | --- | --- | --- | --- | --- | --- |
| JBPS 2-factor model | **–** | **–** | 0.860 | 0.848 | 0.124 | 0.101 |
| JBPS 3-factor model | **–** | **–** | 0.879 | 0.869 | 0.115 | 0.096 |
| JBPS 4-factor model | **–** | **–** | 0.823 | 0.805 | 0.140 | 0.118 |
| JBPS 5-factor model | **–** | **–** | 0.876 | 0.862 | 0.118 | 0.101 |

**Caption:**
Model fit indices (CFI, TLI, RMSEA, SRMR) for the 2- to 5-factor models of the Japanese version of the Boredom Proneness Scale (JBPS) estimated with the **weighted least-squares mean and variance adjusted (WLSMV)** estimator after multiple imputation. Overall, model fit patterns were consistent with those obtained from the complete-case analyses, supporting the robustness of the factorial structure to missing-data handling. Comparative fit indices (CFI/TLI) were generally higher under WLSMV estimation compared with MLR, while the overall factorial structure remained consistent across estimators.

**Supplementary Table S11. Model goodness-of-fit in the confirmatory factor analysis for previous reported short versions of BPS**

|  | GFI | AGFI | CFI | TLI | RMSEA | SRMR |
| --- | --- | --- | --- | --- | --- | --- |
| Ahmed, et al. 1990 (2-factor, 27 item) | 0.738 | 0.693 | 0.583 | 0.547 | 0.105 | 0.133 |
| Vodano, et al. 1990 (5-factor, 27 item) | 0.797 | 0.756 | 0.699 | 0.664 | 0.089 | 0.099 |
| Vodano, et al. 1997 (8-factor, 25 item) | 0.854 | 0.808 | 0.763 | 0.712 | 0.084 | 0.095 |
| Gordon, et al. 1997 (2-factor, 22 item) | 0.668 | 0.596 | 0.531 | 0.479 | 0.125 | 0.138 |
| Gana, et al. 1998 (2-factor, 26 item) | 0.639 | 0.575 | 0.493 | 0.447 | 0.115 | 0.122 |
| Vodanovic, et al. 2005 (2-factor, 12 item) | 0.909 | 0.866 | 0.770 | 0.713 | 0.097 | 0.075 |
| Melton, et al. 2009 (2-factor, 23 item) | 0.806 | 0.766 | 0.710 | 0.679 | 0.096 | 0.097 |
| Craparo, et al. 2013 (3-factor, 16 item) | 0.868 | 0.823 | 0.767 | 0.723 | 0.100 | 0.097 |
| Struk, et al. 2015 (2-factor, 8 item ) | 0.805 | 0.631 | 0.542 | 0.324 | 0.209 | 0.142 |
| Kubel, et al. 2020 (3-factor, 28 item ) | 0.854 | 0.829 | 0.775 | 0.755 | 0.076 | 0.084 |
| Sung, et al. 2021 (2-factor, 7 item) | 0.978 | 0.953 | 0.916 | 0.864 | 0.075 | 0.050 |
| Sung, et al. 2021 (2-factor, 9 item) | 0.952 | 0.916 | 0.848 | 0.789 | 0.090 | 0.066 |

Notes, BPS: boredom proneness scale

Reference range of acceptable goodness-of-fit (GFI: 0.95, AGFI: 0.95, CFI:0.90, TLI: 0.90, RMSEA: 0.06, SRMR: 0.08)

**Supplementary Table S12. Single item statistics of the s-BPSO in the second survey**

| Item | Mean | SD | Skew | Kurtosis | Item Difficulty | Item Discrimination | Item Scalability Coefficient (H) |
| --- | --- | --- | --- | --- | --- | --- | --- |
| bps4 | 2.4 | 1.32 | 0.84 | 0.07 | 0.34 | 0.58 | 0.45 |
| bps14 | 3.03 | 1.48 | 0.47 | -0.48 | 0.43 | 0.6 | 0.45 |
| bps16 | 2.79 | 1.42 | 0.55 | -0.4 | 0.4 | 0.68 | 0.50 |
| bps7 | 3.63 | 1.51 | 0.34 | -0.56 | 0.52 | 0.41 | 0.33 |
| bps8 | 2.93 | 1.28 | 0.72 | 0.38 | 0.42 | 0.53 | 0.41 |
| bps13 | 3.37 | 1.24 | 0.36 | -0.07 | 0.48 | 0.58 | 0.44 |

Mean inter-item-correlation=0.408, Cronbach's α=0.803, Omega Total was 0.84, and Omega Hierarchical was 0.65

Q3 residual statistics (Min: -0.559, First quartile: -0.256, Median: -0.054, Mean: -0.132, Third quartile: 0.045, Max: 0.105)

Reference range of acceptable score (Mean inter-item-correlation 0.15–0.50, Cronbach’s α 0.70–0.95, Item scalability coefficients > 0.3, mean values of Q3 residuals < 0.2)

**Figure S1.** Item Characteristics Curve for each item in s-BPSO

**
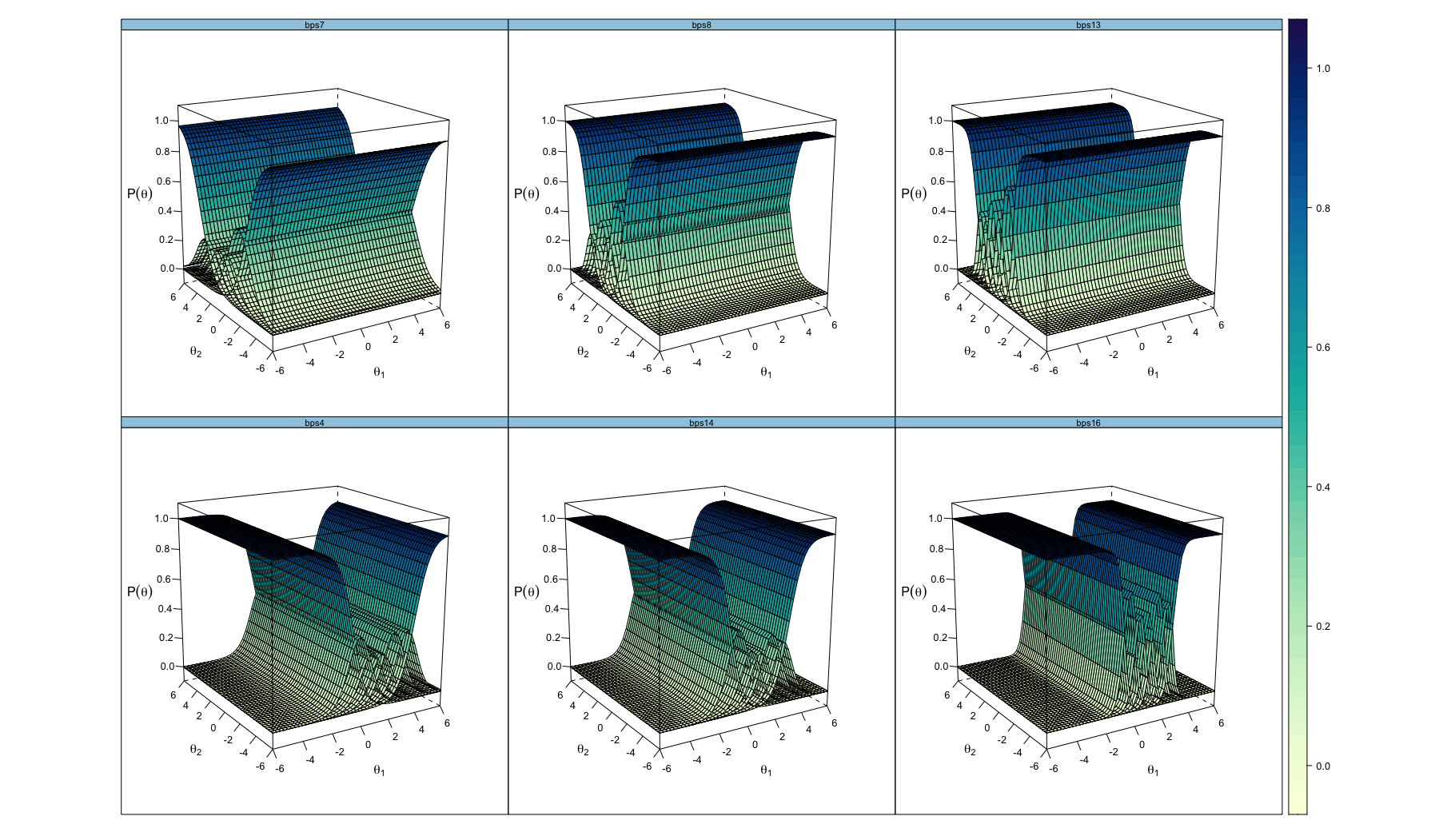
**

*θ1*: latent trait factor 1 (forward-worded items), *θ2*: latent trait factor 2(reverse-worded items)

**Figure S2.** Test Information Function Plot of s-BPSO


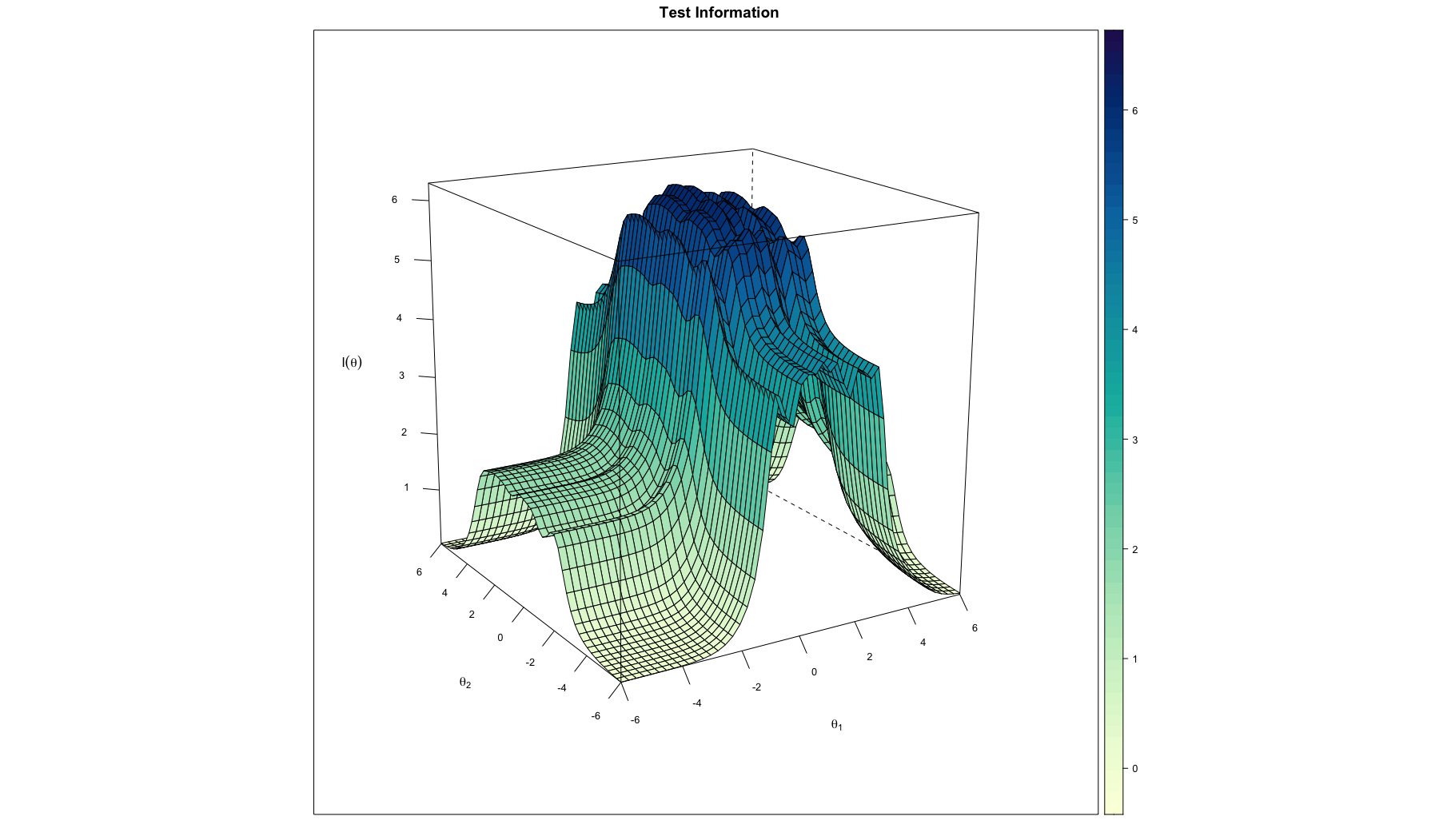


*I(θ):* Test Information Function, *θ1*: latent trait factor 1 (forward-worded items), *θ2*: latent trait factor 2(reverse-worded items)

**Figure S3.** Structural diagram of the confirmatory analysis assuming two-factor model for the s-BPSO using the second survey


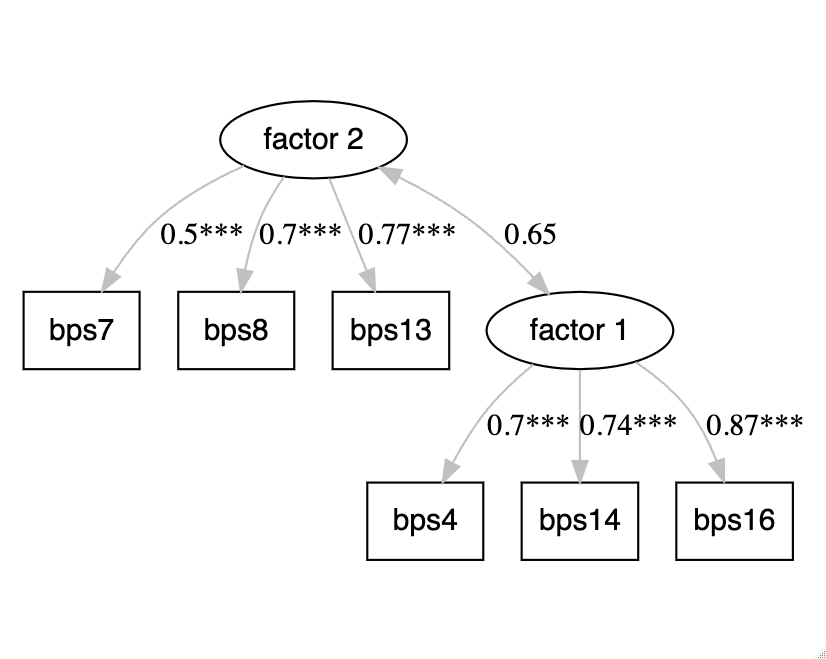


factor 1: latent trait factor 1 (forward-worded items), factor 2: latent trait factor 2 (reverse-worded items)

The standardized estimated coefficient is shown for each path.

***: *P* values less than 0.001.


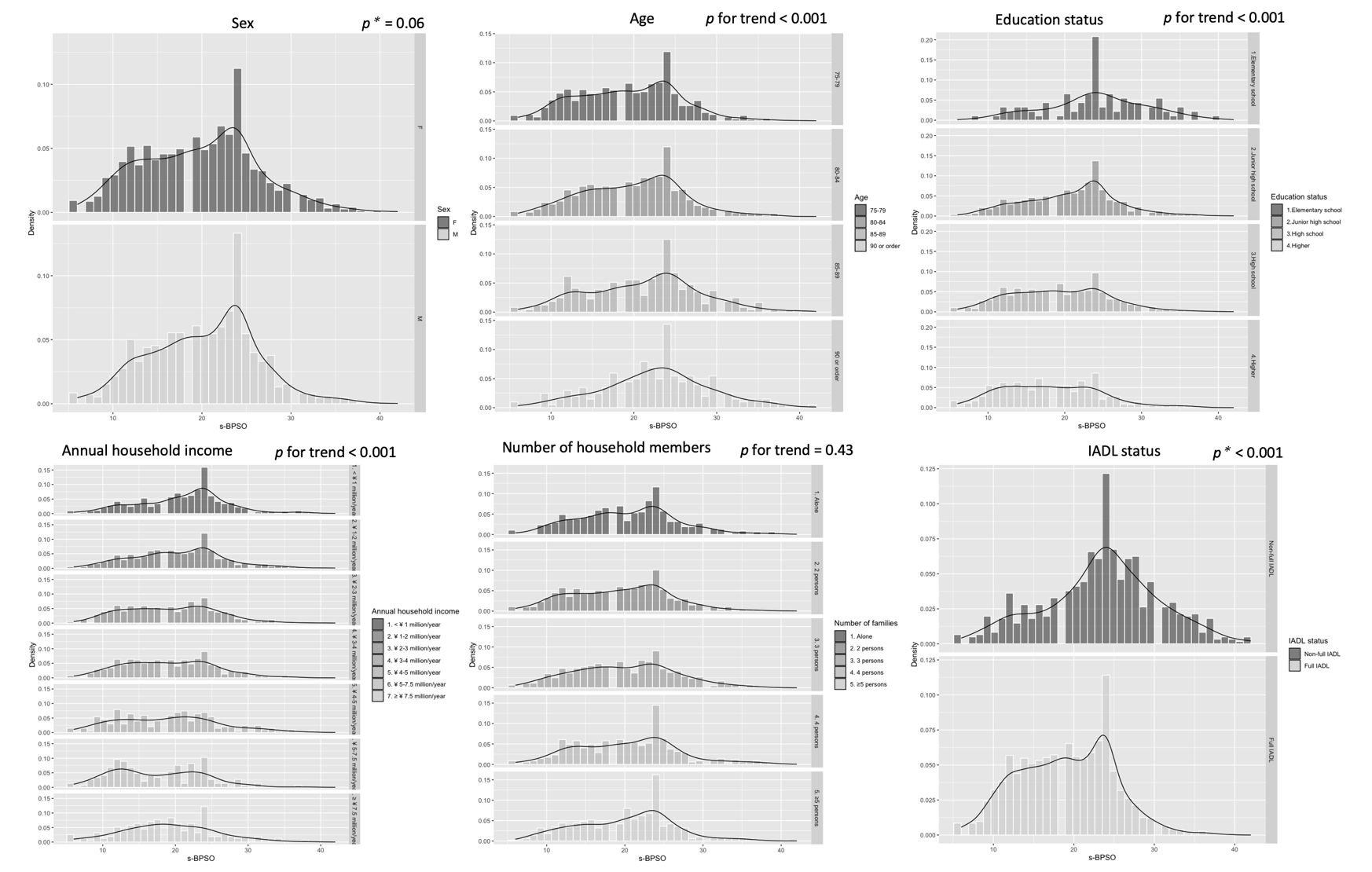
**Figure S4.** The distribution of s-BPSO across socioeconomic factors among community dwelling older population

*p** values calculated using *t* test. The other *p* for trend values were calculated by Jonckheere-Terpstra trend test.
